# Modeling, Simulation and Implementation of All Terrain Adaptive Five DOF Robot

**DOI:** 10.3390/s22186991

**Published:** 2022-09-16

**Authors:** Zhe Wang, Jianwei Zhao, Gang Zeng

**Affiliations:** School of Mechanical Electronic & Information Engineering, China University of Mining and Technology-Beijing, Beijing 100089, China

**Keywords:** robot, all terrain, Adams, simulation, Ansys, arthropods, obstacle-crossing

## Abstract

The ability of an off-road robot to traverse obstacles determines whether the robot can complete complex environmental tasks. In order to improve the off-road ability of off-road robots, this paper proposes a new design idea, in which four hub motors are the power system of the robot, the steering system of the robot is composed of a steering machine and a stepping motor, and a five degree of freedom robot model is established. The body structure is designed according to the characteristics of arthropods. The body structure is divided into three modules, and the connecting rod is used as the joint system of the robot to connect the three parts. The body can deform when facing complex obstacles, so as to adapt to different terrains. Then the body structure is simplified, and a mathematical model is established to describe the mathematical relationship between body joint changes. In order to verify the ability of the adaptive all-terrain cross-country robot to traverse obstacles, the load-bearing experiment and obstacle-crossing simulation experiment were carried out through Adams software, and the continuous traversing performance at low obstacles and the ability to break through high obstacles were tested, respectively. The experimental results prove that the designed adaptive all-terrain off-road robot is feasible, has good carrying capacity, and has good passability in the face of low obstacles and high obstacles. Using Ansys software to perform finite element analysis on the wheel connection, the experimental results show that the strength meets the material strength requirements. Finally, a real vehicle test is carried out to verify the correctness of the simulation results.

## 1. Introduction

In order to reduce the workload of people, robots have become a good helper for people, and can help people complete dangerous and heavy tasks. Mobile service robots are one of the research hotspots among many robots, and their application range is also very wide, which is gradually expanding to space exploration [1], military reconnaissance, explosives disposal and rescue [2], entertainment services, and other fields [3].

A lot of research has been done on ground mobile robots, and a lot of efforts have been made [4]. Ground mobile robots include wheeled robots, legged robots, and crawler robots. Wheeled robots can simply and effectively move at high speed and stably on flat and complex roads or sloping terrain [5], but in unstructured environments, the use of legged robots and crawler robots is also a valuable choice. Crawler robots can move on rugged terrain, because their contact surface with the ground is much larger than wheeled and legged robots [6], which can make the operation more stable, but they usually move at a slower speed and are less energy efficient. Legged robots have good obstacle-crossing performance, such as big dog robots [7]. However, due to the complex structure and high integration of legs, its cost is also very high. Especially for robots with dynamic gait planning, because the high complexity of dynamic gait is not only related to control, but also related to mechanical structure. Therefore, reducing the number of legs can simplify its complexity and reduce its cost [8]. With the further improvement of performance requirements, research on hybrid robots has appeared [9]. At present, hybrid robots mainly include wheel-foot hybrid robot (WLHR), wheel-track hybrid robot (WTHR), wheel-track-leg hybrid robot (WTLHR), etc. [10]. These robots improve the obstacle-crossing ability by changing the way of walking.

In order to adapt to different terrains, wheeled robots are usually integrated with other mechanical equipment. A. Alamdari et al. [11] proposed an alternative articulation mechanism for the leg-wheel subsystem and presented a general framework and method for kinematic-static design optimization for planar rough terrain traversal tasks. J. Xiao et al. [12] designed a wheeled climbing robot with an eddy current rotor that can provide adhesion from differential pressure. Y. Takita et al. [13] proposed an eight-wheeled robot, the shape of the body is deformable due to the suspension system. Ref. [14] developed an autonomous upright stair-climbing mobile robot application for indoor service. The robot can rotate a pair of triangular modules to climb stairs while maintaining balance. A. Spherical mobile robot that rolls on a plane with the help of two internal rotors and works according to the principle of conservation of angular momentum, studied by V.A. Joshi [15]. Ni, Liwei et al. studied the attitude control performance of wheel-legged robots (WLR) on uneven terrain and proposed a novel four-wheel-legged robot (FWLR) with tandem slow-speed active suspension system (SSASS) [16]. However, wheeled robots are better at simply and effectively moving stably at high speed on flat and complex roads or sloping terrain [5], but they do not perform satisfactorily in some uneven and complex environments, such as trenches, stairs, and so on.

Multipedal robots, including bipeds, quadrupeds, and hexapods, have attracted much attention in robotics and biomedicine due to their great potential in robotics. BigDog from Boston Dynamics [7] is probably the most famous multi-legged robot in recent years. Over the years, it has imitated the mobility, autonomy, and speed of living things. As for bipeds, Honda’s success [17] sparked worldwide research on anthropomorphic robots [18], and Boston Dynamics produced a map of bipedal anthropomorphic robots that can even perform backflips. S. Jin et al. of Stanford University designed a bionic climbing robot that is also a quadruped capable of scaling smooth vertical surfaces. The robot, called Stickbot, uses sticky material to climb like a gecko [19]. Marko Bjelonic et al. designs hexapod robots for use in unstructured terrain [20]. Rizuwana Parween et al. [21] developed a self-configuring hybrid robot called Tarantula II. The platform is a quadruped robot with hybrid motion and reconfiguration capabilities to achieve variable height and width. G. Gogu et al. pointed out that the formula for fast calculation of mobility is not applicable to many classical mechanisms and proposed a new formula for fast calculation of mobility applicable to any parallel mechanism [22]. A. Cully et al. developed an intelligent trial-and-error algorithm that enables a multipedal robot to adapt to impairments in its environment and it takes less than two minutes in a large search space without self-diagnosis or contingency planning, demonstrating robust adaptation when some of the multipedal robot’s legs are damaged potential [23]. Multipedal robots have many advantages and can even do some things animals do, but controlling them is very complex, especially for bipedal robots, which may not walk properly on certain terrains such as sand. Snake robots imitate the movement of snakes or inchworms with different gaits such as accordion, lateral bending, inchworm/caterpillar, climbing, and lateral waves [24]. Z.Y. Bayraktaroglu [25] studied wheelless serpentine motion, an inchworm-like motion, through lateral fluctuations, and these experiments showed that the design of the controller depends on the mechanical structure and the motion environment of the serpent robot is highly valued. A. A. Transeth et al. [26,27] explored obstacle-assisted mobility for snake robots. They built a non-smooth 3D model of the snake robot with external obstacles and verified the simulation results by comparing the link positions in the simulation and experiments. C. Wright et al. [28] designed a unified modular snake robot that can climb trees relative to the drive axis of the preceding module, around the spine of the robot, since the drive axis of each module is rotated 90 degrees. Combining the snake robot with passive wheels, T. Kamegawa et al. [29] developed a snake robot that can achieve a spiral cylindrical climbing motion. Snake robots can be used in many situations and even climb pipes [30], but they suffer from shortcomings in speed and efficiency, which have been a challenge in application. Unlike crawlers and leg mechanisms, deformable robots can move on rough terrain through deformation. S. Hirai et al. [31,32,33,34] describe the principle of robots crawling and jumping through deformation, i.e., gravitational potential energy. Not only did they provide a strategy for rolling robots, but they also built several prototypes to validate it, including shape memory alloy-driven circular soft robots, spherical prototypes, and tensegrity monoliths. First proposed in *Architecture* [35], a tensegrity is a mechanical structure consisting of a set of rigid elements connected by elastic tensegrity elements. The tensegrity monolithic robot structure maintains its shape due to the balance of forces and, thus, can deform to adapt to the terrain. Its kinematics and statics were analyzed [36]. Y. Koizumi and S. Hirai et al. [35,37] constructed a large-scale tensegrity robot driven by pneumatic soft actuators, formulated its geometry, proposed some rolling strategies, and simulated them with a dynamic model rolling process. The Dynamic Tension Robotics Laboratory at NASA Ames Research Center has developed a software toolkit, called the NASA Tension Robot Toolkit (NTRT), to analyze, simulate, and design tension robots. They built a superball for planetary exploration, and it has been shown to be particularly suitable for high-state trajectory tracking [38]. K. Kim et al. [1] described the design and control of a rapid prototyping tensegrity robot for locomotion using the NTRT toolkit. Fei Zhang et al. [39] proposed a deformable polygon robot with multiple joints, which can adapt to different 2D terrains and move angularly quickly with a simple structure. Transformable robots promise terrain adaptation, but common structures are often cumbersome. Although the wheeled legged robot improves its ability to adapt to uneven and complex environments, its bearing capacity is much worse than that of a wheeled robot. This paper mainly studies how to improve the ability of a wheeled robot to deal with uneven and complex environment while maintaining its high carrying capacity. By redesigning the body structure, it can adapt to uneven and complex terrain, and has the high bearing performance of wheeled robots.

The robot designed in this paper is mainly used in scenarios where the road is rugged and the traffic is difficult after disasters such as earthquakes or forest fires, and large transport vehicles cannot be used to transport rescue equipment. The robot is designed to be of moderate size, easy to disassemble and spliced, and can travel freely in rugged environments. At the same time, it can load rescue equipment and rescue materials, transport rescue equipment for a team of rescuers, and reduce the physical consumption of rescuers when they are not in rescue. According to the working environment of the robot, a design scheme of a new wheeled off-road robot that is more flexible, has stronger ability to overcome obstacles and has a certain carrying capacity is proposed. The robot uses a special link-spring joint system to connect the front, body, and rear of the car. Compared with the above literatures [5,13,14], the robot design method is simple, and the structure is improved on the four-wheeled wheeled robot, which does not require too many wheels [13] and complex structures [14]. At the same time, it has a strong carrying capacity. Through the change of the link-spring joint angle and the independent suspension system, it can adapt to various complex environments.

The robot adopts a modular design concept, and the whole is decomposed into three parts: the front end (steering control), the middle end (loading connection), and the tail end (supporting and pushing). Two adjacent parts are connected by joints. Suspension systems are arranged at the front and rear to imitate the appendages of arthropods, which can automatically adapt to various terrain environments. The front end is equipped with steering gear steering system to control the steering of wheeled robots. At the same time, the front and rear are equipped with detachable support plates, which are convenient for supporting the motor drive and the expansion of more functions. The middle part is composed of a symmetrical frame, and the front side and the rear side are connected by hinges and bolts, which is convenient for the separation and folding of the vehicle and makes the vehicle easy to carry.

## 2. Mechanical Structure Design

### 2.1. Steering System

In the design of wheeled robots in the past, the steering method usually uses differential steering [40], and the steering function is realized using the speed difference of four wheels. However, in the actual application process, when the load of the vehicle is large, the speed control of each wheel fails, and the steering function can no longer be realized. Therefore, the vehicle adopts the steering system of steering gear, which is composed of gear and rack. The motor is used to drive the gear to rotate, and then the force is transmitted to the rack through the meshing of the gear and the rack. The two ends of the rack are connected with the horn at the fixed part of the wheel through the connecting rod, and the connecting rod is used to push and pull the wheel, so that the vehicle can turn.

### 2.2. Suspension System

This vehicle adopts double wishbone independent suspension [41], which is composed of upper control arm, lower control arm, and shock absorber. The upper control arm is U-shaped, the lower control arm is triangular, and the upper control arm is shorter than the lower control arm. The upper control arm and the lower control arm are connected to the steering horn and the front frame, and the shock absorber is connected to the lower control arm and the front frame. Through multi-node connection, the integrity of the upper and lower control arms and the front body can be improved. The up and down movement of the wheels can be achieved through the upper and lower control arm, and the kinetic energy of the movement is transferred to the damper through the spring on the shock absorber. By adjusting the damper to adjust the vibration amplitude, the stability of the vehicle motion is maintained. The suspension system can change the position of each wheel to automatically adapt to uneven terrain.

### 2.3. Joint System

The vehicle is divided into three parts: front, middle, and rear, and the two adjacent parts are connected by joints. The joint system of this vehicle is composed of eight connecting rods and four shock absorbers, and the two sides of the connecting rod are spherical heads. It is symmetrically distributed in the front end and middle end, middle end, and tail end. The connecting rods are arranged in two layers; the distance between the lower layer connecting rods is greater than the distance between the upper layer connecting rods, and the overall arrangement is in a regular trapezoid shape. The shock absorber connects the front body and the third layer of the middle frame. Through this joint system, the front, middle, and rear parts are connected as a whole. When obstacles are encountered, the push-pull effect between them is used to make the vehicle deform automatically to adapt to the environment. At the same time, the load-bearing capacity and support stability of the vehicle body can be improved.

### 2.4. Drive System

The four wheels are independently driven by in-wheel motors. The speed of each wheel can be controlled separately, and the body shape can be changed by changing the speed of different wheels to pass different obstacle terrains.

**Figure 1:**
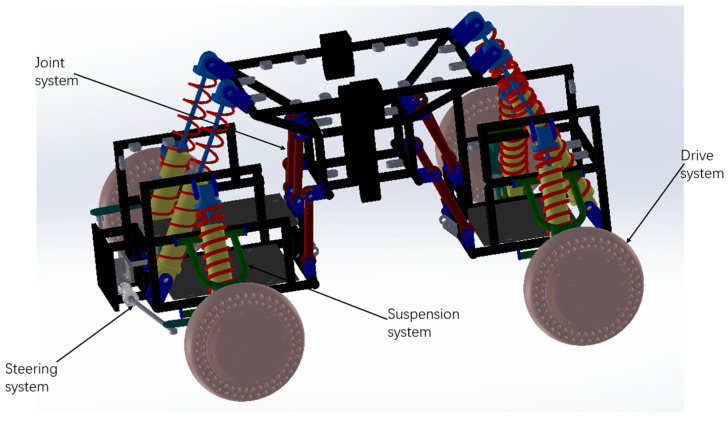
Vehicle structure.

Figure 1 is a three-dimensional view of the vehicle structure.

## 3. Mathematical Model

### 3.1. Forward Kinematics Analysis

When the vehicle encounters obstacles, it adapts to various terrain environments by adjusting the posture of the body, and the posture change of the body mainly depends on four rotational joints. By establishing a kinematic model of the vehicle body, it can be used as a basis for analyzing the body posture changes. The vehicle is composed of the front body, the middle frame, and the rear body, which are connected by connecting rods. The distribution of connecting rods is symmetrical in the front and rear, and the position is determined under the combined action of eight connecting rods. Therefore, when the mathematical model is established, the entire vehicle mechanism can be simplified to obtain a simplified model as shown in the figure. The simplified model consists of five connecting rods, which are connected to each other through a rotating pair. While all the connecting rods move in the same plane, the kinematics analysis can be simplified to a plane positive kinematics problem.

**Figure 2:**
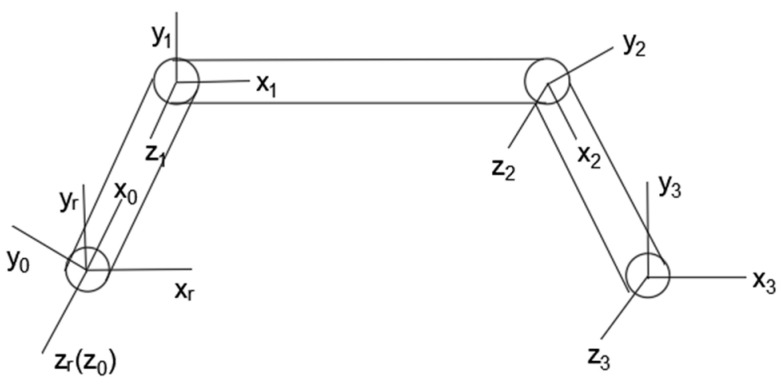
Simplified vehicle model.

Based on the simplification and analysis of the model, the D-H method is used to model the body kinematics [42], as shown in the Figure 2.

The D-H method uses four kinematics parameters to describe the attitude of each link. The length of the connecting rod and the angle α represent the parameters of the connecting rod itself, and the offset d and the joint angle θ represent the relative relationship between the adjacent two connecting rods. A coordinate system for the rotation center of the connection between the front body and the connecting rod is established, and a right-handed coordinate system with the z-axis direction outward along the axis direction is established. The right-handed coordinate systems {0}, {1}, and {2} are established at joints 0, 1, and 2, and the right-handed coordinate system {3} is established at the rotational center of the connection between the rear body and the connecting rod, where the axes of all joints are parallel. The D-H parameters of the vehicle are shown in Table 1. 

Among them, $a_{1}$, $a_{2}$, $a_{3}$ represent the length of the front connecting rod, the length of the middle frame, and the length of the rear connecting rod.

Through homogeneous transformation matrix of adjacent connecting rod:

(1) $${}_{i}^{i-1}T=\left[\begin{array}{cccc} \cos\theta_{i} & -\sin\theta_{i}\cos\alpha_{i} & \sin\theta_{i}\sin\alpha_{i} & a_{i}\cos\theta_{i}\\ \sin\theta_{i} & \cos\theta_{i}\cos\alpha_{i} & -\cos\theta_{i}\sin\alpha_{i} & a_{i}\sin\theta_{i}\\ 0 & \sin\alpha_{i} & \cos\alpha_{i} & d_{i}\\ 0 & 0 & 0 & 1 \end{array}\right]$$

The positive kinematics equation of the rear body coordinate system {3} relative to the {r} coordinate system can be obtained:

(2) $${}_{3}^{r}T=\left[\begin{array}{cccc} \cos(\theta_{0}+\theta_{1}+\theta_{2}+\theta_{3}) & -\sin(\theta_{0}+\theta_{1}+\theta_{2}+\theta_{3}) & 0 & p_{x}\\ \sin(\theta_{0}+\theta_{1}+\theta_{2}+\theta_{3}) & \cos(\theta_{0}+\theta_{1}+\theta_{2}+\theta_{3}) & 0 & p_{y}\\ 0 & 0 & 1 & 0\\ 0 & 0 & 0 & 1 \end{array}\right]$$

(3) $$p_{x}=a_{1}\cos(\theta_{0}+\theta_{1})+a_{2}\cos(\theta_{0}+\theta_{1}+\theta_{2})+a_{3}\cos(\theta_{0}+\theta_{1}+\theta_{2}+\theta_{3})$$

(4) $$p_{y}=a_{1}\sin(\theta_{0}+\theta_{1})+a_{2}\sin\left(\theta_{0}+\theta_{1}+\theta_{2}\right)+a_{3}\sin\left(\theta_{0}+\theta_{1}+\theta_{2}+\theta_{3}\right)$$

### 3.2. Inverse Kinematic Analysis

In the process of movement, when the vehicle encounters obstacles, the speed of the front body will slow down, and the rear body will continue to move forward to push the front body across the obstacles. In this process, because the relative position of the front body and the rear body changes, $\theta_{0}$ and $\theta_{3}$ will change. In order to make the vehicle body reach the equilibrium state, $\theta_{1}$ and $\theta_{2}$ will also produce corresponding change angles, so that the whole vehicle will deform and adapt to the terrain. When performing inverse kinematics calculations, assuming that the posture and position parameters of the rear body coordinate system are known, the inverse kinematics analysis needs to solve the values of $\theta_{0}$, $\theta_{1}$, $\theta_{2}$, and $\theta_{3}$.

(5) $${}_{3}^{r}T=\left[\begin{array}{cccc} n_{x} & o_{x} & a_{x} & p_{x}\\ n_{y} & o_{y} & a_{y} & p_{y}\\ n_{z} & o_{z} & a_{z} & p_{z}\\ 0 & 0 & 0 & 1 \end{array}\right]{=}_{0}^{r}T_{1}^{0}T_{2}^{1}T_{3}^{2}T$$

This paper uses the method of separating variables to solve the problem, and the final results are as follows:

(6) $$\theta_{0}=\arccos(n_{x}\cos(\theta_{1}+\theta_{2}+\theta_{3})-o_{x}\sin(\theta_{1}+\theta_{2}+\theta_{3}))$$

(7) $$\theta_{1}=\arctan\frac{n_{z}\cos(\theta_{2}+\theta_{3})-o_{z}\sin(\theta_{2}+\theta_{3})}{o_{z}\cos(\theta_{2}+\theta_{3})+n_{z}\sin(\theta_{2}+\theta_{3})}$$

(8) $$\theta_{2}=\arctan(\frac{n_{z}\cos\theta_{3}-o_{z}\sin\theta_{3}}{o_{z}\cos\theta_{3}+n_{z}\sin\theta_{3}})$$

(9) $$\theta_{3}=\arctan(\frac{n_{z}}{o_{z}})$$

## 4. Adams Simulation Analysis

**Figure 3:**
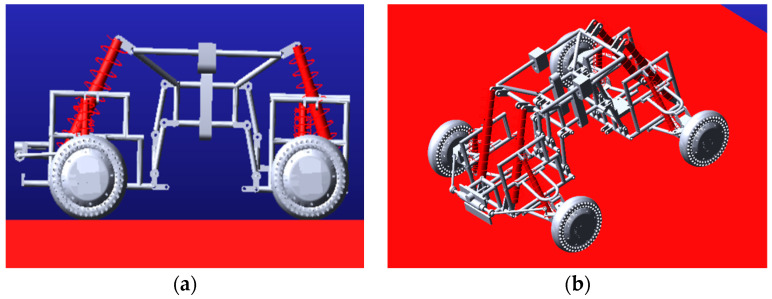
The vehicle model was imported into Adams. (**a**) is front view, (**b**) is oblique view, easy to observe the structure of the vehicle.

In the design process of the whole vehicle, a complete assembly model was established in SolidWorks. The solid model was imported into Adams, and the kinematics simulation analysis of the vehicle was carried out by Adams software. After importing the entity model, the name of each constituent entity was modified, and then the material properties of each entity were modified. The main material of this vehicle is aluminum (Density: 2740 kg/m^3^, Young’s modulus: 72 Gpa, Poisson ratio: 0.33). After the model was imported into Adams, all the coordination in SolidWorks failed, and the kinematic pair needed to be added again. At this point, the preparatory work before the simulation was completed. Figure 3 shows the effect after the vehicle model is imported into Adams.

### 4.1. Statics Analysis (Loading Experiment)

**Figure 4:**
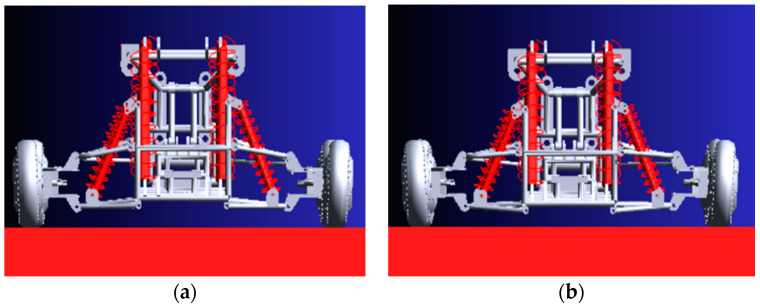
Vehicle load comparison chart. (**a**) The body is not loaded, (**b**) The body is loaded.

According to the design requirements of the vehicle, the vehicle can carry 200 kg (200 × 9.8 = 1960 N). In order to facilitate the simulation experiment, a vertical downward force of 1000 N was applied to the left and right sides of the middle frame, respectively. The experimental results are shown in Figure 4.

**Figure 5:**
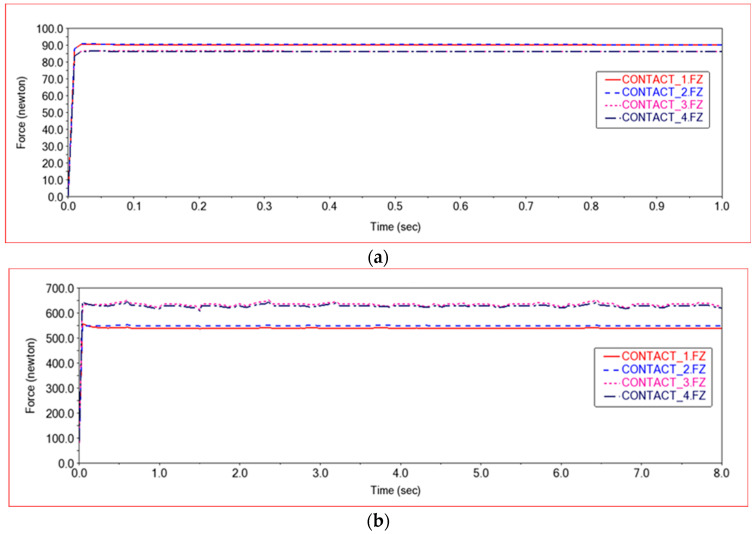

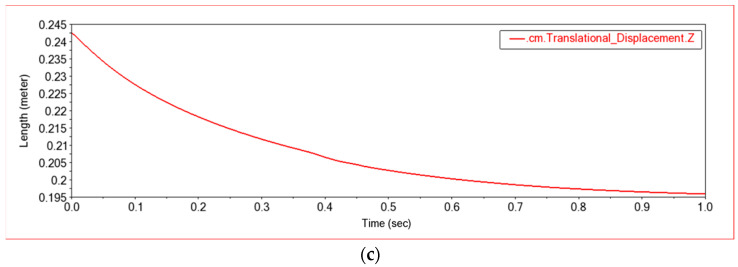
Vehicle load test experiment. (**a**) Supporting force curve before loading, (**b**) Supporting force curve after loading, (**c**) Centroid displacement curve. CONTACT_1—Left front wheel and ground contact force; CONTACT_2—Right front wheel and ground contact force; CONTACT_3—Left rear wheel and ground contact force; CONTACT_4—Right rear wheel and ground contact force.

Figure 4a is the state diagram of the vehicle body when the vehicle is only self-weight (rear view). Figure 4b is the state diagram of the vehicle body after applying 2000 N (rear view). It can be seen that the vehicle body sinks after the force of 2000 N, but it is still within the acceptable range. Figure 5a shows the curve of the contact force between the four wheels and the ground without load. It can be seen from Figure 5a that the contact force between the left front wheel and the ground is equal to the contact force between the right front wheel and the ground. The contact force of the left rear wheel with the ground is equal to the contact force of the right rear wheel with the ground. The contact force between the front wheels and the ground is slightly greater than the contact force between the rear wheels and the ground. Because the front frame is equipped with a steering mechanism, the force of the front wheels is slightly greater than that of the rear wheels when it is not loaded.

Figure 5b is the curve of the contact force between the four wheels and the ground after applying a force of 2000 N. Within a short time after the start of the simulation, the vehicle body regained its equilibrium state. The contact force of the left front wheel with the ground is equal to the contact force of the right front wheel with the ground. The contact force of the left rear wheel with the ground is equal to the contact force of the right rear wheel with the ground. Because the front frame needs to be equipped with steering mechanism and steering motor in the design, the front frame is longer than the rear frame in terms of design size. As a result, the angle between the front shock absorber and the ground is smaller than the angle between the rear shock absorber and the ground. After bearing the load, the rear frame will bear more force, so the contact force between the two rear wheels and the ground is greater than the contact force between the two front wheels and the ground. Figure 5c shows the change curve of the mass center position of the rear frame during the process of bearing a load of 2000 N. At the beginning of the simulation, the mass center dropped quickly. As the position dropped, the shock absorber was compressed, slowing down the decline, and finally reached a stable state of equilibrium.

### 4.2. Dynamic Simulation

The ability of the adaptive all-terrain wheeled off-road robot to cross obstacles determines the performance of the robot. Therefore, it is necessary to carry out obstacle-crossing simulation experiments for it. In this paper, Adams software is used to simulate the dynamics of the vehicle. The dynamic analysis of the steering mechanism is not considered in the simulation. When the kinematic pair is added, the steering mechanism is fixed with the front frame, and the linear motion of the vehicle is only considered.

#### 4.2.1. Terrain Construction

**Figure 6:**
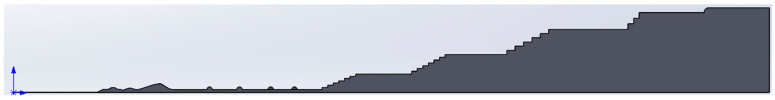
Obstacle crossing experiment terrain.

The terrain of the vehicle’s obstacle crossing experiment was established through SolidWorks, and then imported into Adams, merged with the vehicle model in Adams, and used as the vehicle’s experimental platform. The terrain has a total length of 27 m, a height of 3 m, and a width of 2 m. It is mainly composed of rugged slopes, a semi-circular convex platform, and steps. The rugged slope shape is drawn and stretched by spline curves, which are drawn randomly to imitate the unknowns of rugged roads. The semi-circular boss is drawn using a semi-circular arc with a radius of 10 cm, and the stretch length is 1 m. There are two bosses on the left and right sides to test the ability of the vehicle to cross obstacles when it encounters obstacles on one side. In order to fully reflect the vehicle’s obstacle crossing performance, seven steps of 20 × 8 (cm), seven steps of 20 × 10 (cm), six steps of 30 × 15 (cm), third steps of 20 × 20 (cm), and a first-step arc step with a radius of 30 cm are designed in the terrain. These steps are the main test of the vehicle’s low obstacle continuous climbing and high obstacle key breakthrough performance. The terrain is shown in Figure 6:

#### 4.2.2. Obstacle Crossing Experiment 1

**Figure 7:**
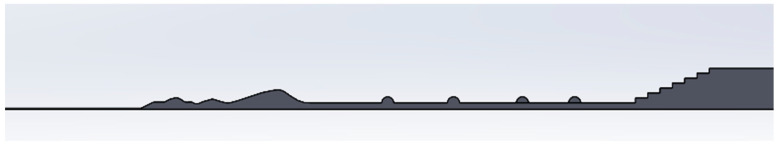
The first section of the experimental terrain.

**Figure 8:**
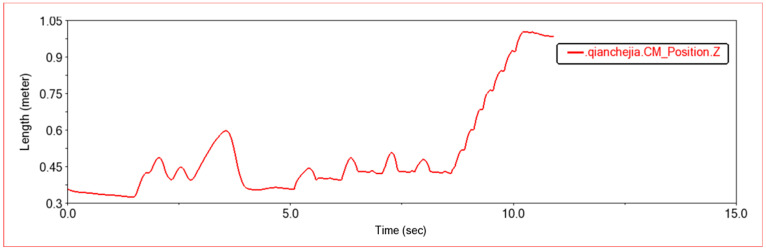
The trajectory diagram of the center of mass of the front frame.

**Figure 9:**
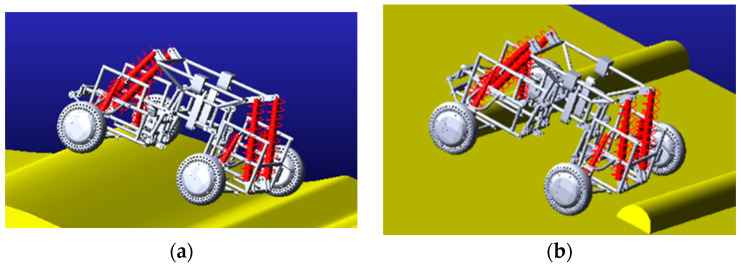

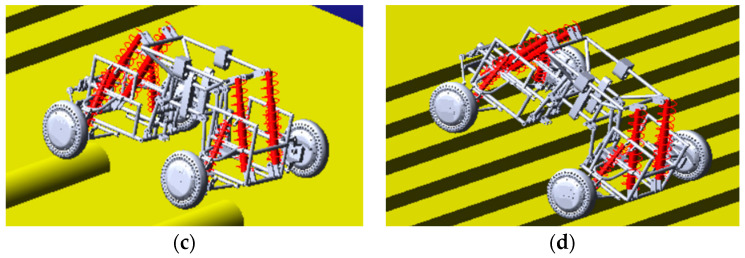
Animation simulation of obstacle crossing experiment. (**a**) Car driving through rough road, (**b**) The right suspension passes through the boss, (**c**) The left side suspension passes through the boss, (**d**) Car climbs up the steps.

The terrain conditions of this experiment are rugged slopes, semi-circular bosses distributed on the left and right sides, and seven steps of 20 × 8 (cm) steps. The main test is the ability of the vehicle to continuously cross obstacles under low obstacle conditions. The terrain is shown in Figure 7:

Figure 8 is a curve showing the change of the center of mass of the front frame on the z-axis with time during this experiment. It can be seen that the change trend of the center of mass of the front frame on the z-axis is exactly the same as that of the original terrain design. The curve is continuous and smooth. When crossing low obstacles, the movement is smooth, and its movement meets the design requirements. Figure 9 is the animation simulation of vehicles crossing various obstacles.

**Figure 10:**
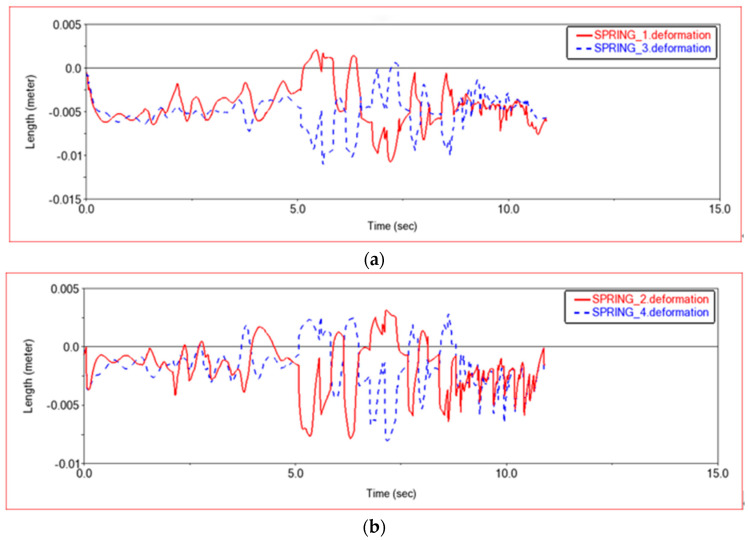
Deformation diagram of suspension shock absorber. (**a**) Deformation diagram of front suspension shock absorber, SPRING-1-Left front suspension shock absorber, SPRING-3-Right front suspension shock absorber; (**b**) Deformation diagram of rear suspension shock absorber. SPRING-2-Left rear suspension shock absorber; SPRING-4-Right rear suspension shock absorber.

Figure 10 shows the comparison of the deformation of the left and right front suspension shock absorbers and the comparison of the deformation of the left and right rear suspension shock absorbers in the simulation experiment. It can be seen from Figure 10 that the maximum deformation difference occurs in the middle of the simulation process, that is, when the vehicle passes through the terrain of a single-sided semicircular boss, the left and right suspension shock absorbers show opposite deformations. This is because when the car passes a single-sided semicircular boss, because the boss is very high, the shock absorber on the side in contact with the boss will be compressed and deformed. The side without contact with the boss will be lifted to make the wheel suspended, so the deformation of the suspension shock absorber will be reduced. When climbing steps, in order to adapt to the shape of the steps, the suspension positions on the left and right sides may be different, so the amount of deformation of the shock absorber is slightly different.

**Figure 11:**
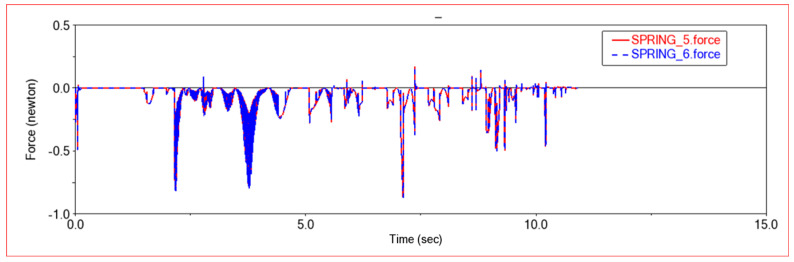
Force diagram of front frame shock absorber. SPRING-5-Front frame left shock absorber; SPRING-6-Front frame right shock absorber.

Figure 11 is the force diagram of the front frame shock absorber. It can be seen that the front frame shock absorber is hardly stressed when moving in a plane. However, when encountering obstacles, the force of the front frame shock absorber will fluctuate frequently. As the picture shows, in this experiment, the force of the shock absorber fluctuates sharply when the vehicle passes through a rugged slope. Because the rugged slopes are drawn randomly using spline curves, the road conditions are very complex. Many different deformations are concentrated in a short distance. In order to adapt to the road conditions, the front shock absorber will frequently vibrate to relieve the impact of the terrain. The front shock absorber mainly plays a role of buffering and damping when crossing obstacles. In order to keep the vehicle body stable when crossing obstacles, a shock absorber with larger damping can be selected as the front frame shock absorber.

**Figure 12:**
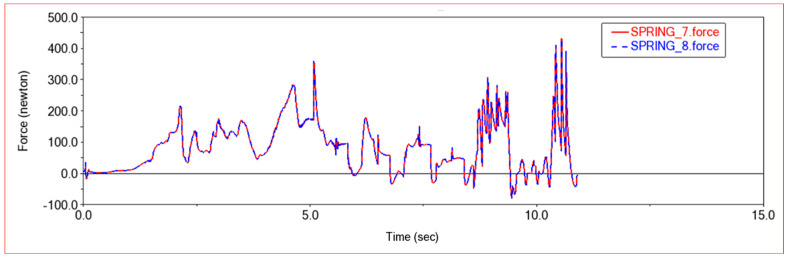
Force diagram of rear frame shock absorber in Experiment 1. SPRING-7-Rear frame left shock absorber; SPRING-8-Rear frame right shock absorber.

**Figure 13:**
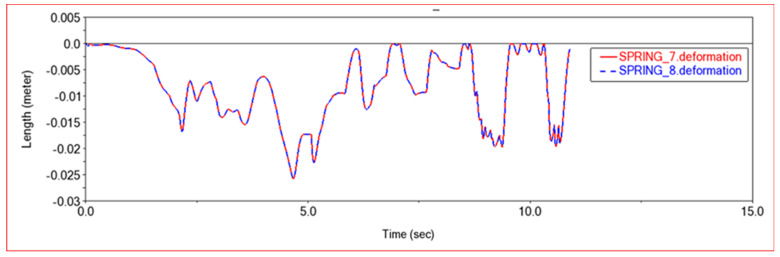
Deformation diagram of rear frame shock absorber in Experiment 1. SPRING-7-Rear frame left shock absorber; SPRING-8-Rear frame right shock absorber.

Figure 12 and Figure 13 are, respectively, the force diagram and deformation diagram of the left and right shock absorbers of the rear frame in this experiment. It can be seen from the above figure that the force and deformation of the left and right shock absorbers of the rear frame are exactly the same, so that when crossing obstacles, the left and right thrusts can be guaranteed to be the same, so that the vehicle can move forward without deviation. It can be seen from the force diagram that when the vehicle encounters an obstacle, the rear frame shock absorber will provide thrust for the body to push the vehicle over the obstacle. As the slope of the obstacle increases, the thrust provided by the rear frame shock absorber increases. When climbing steps, the thrust provided by the rear frame shock absorber is very large at the beginning, the thrust is small in the middle period, and the thrust is increased in the final stage. This is because when the front frame touches the steps and starts to climb the steps, the rear frame shock absorbers will provide thrust to push the front wheels up the steps until the rear wheels also climb the steps. The thrust of the vehicle will be reduced, so that the car climbs the steps into a stable state. After the front wheels climb the steps, the rear wheels still have to climb the steps, so the thrust of the rear frame shock absorber will increase. After pushing the whole vehicle up the steps, the thrust will decrease to zero.

**Figure 14:**
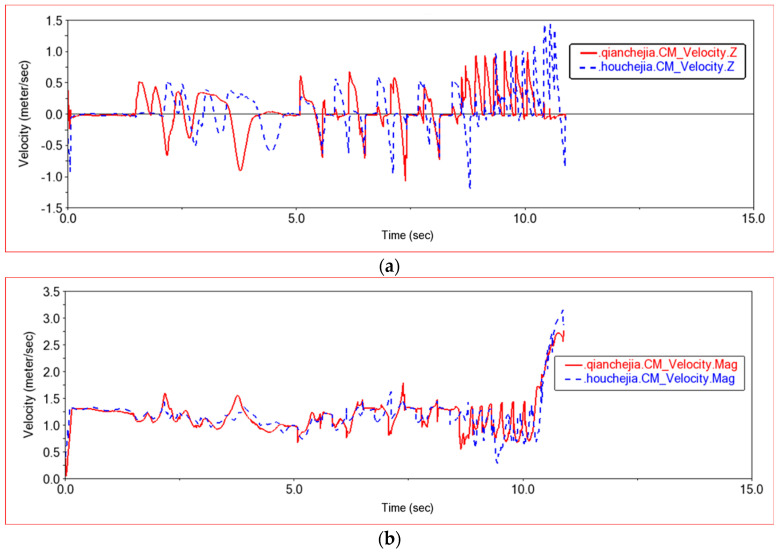
Speed comparison between front frame and rear frame. (**a**) The z-axis speed curve of the front frame and the rear frame, (**b**) The speed curve of the front frame and the rear frame. qianchejia.CM-Front frame center of mass; houchejia.CM-Rear frame center of mass.

As shown in Figure 14, the mass center speed of the front frame and the rear frame is compared in this experiment. When the car crosses obstacles, compared to the plane motion, the speed changes mainly on the z-axis. It can be seen from Figure 14a that the speed of the z-axis is 0 when the car is moving in a plane. When climbing obstacles, the speed of the z-axis increases. As the road surface fluctuates, the speed of the z-axis changes continuously. The middle curve is the speed change curve when the car passes through the semicircular boss. The wheels move along the arc road. When climbing the front semicircle, the speed is first fast and then slow. The middle curve is the speed change curve when the car passes through the semicircular boss. The wheels move along the arc road. When climbing the front semicircle, the speed is first fast and then slow. The third curve is the speed change curve when the car climbs the steps. Because the characteristic of the steps is to move upwards and then move in a plane, there is a sawtooth speed fluctuation when climbing the steps. Because there are seven steps in total, the speed fluctuates seven times. The speed curve of the rear frame is similar to that of the front frame, but lags behind the front frame in time.

**Figure 15:**
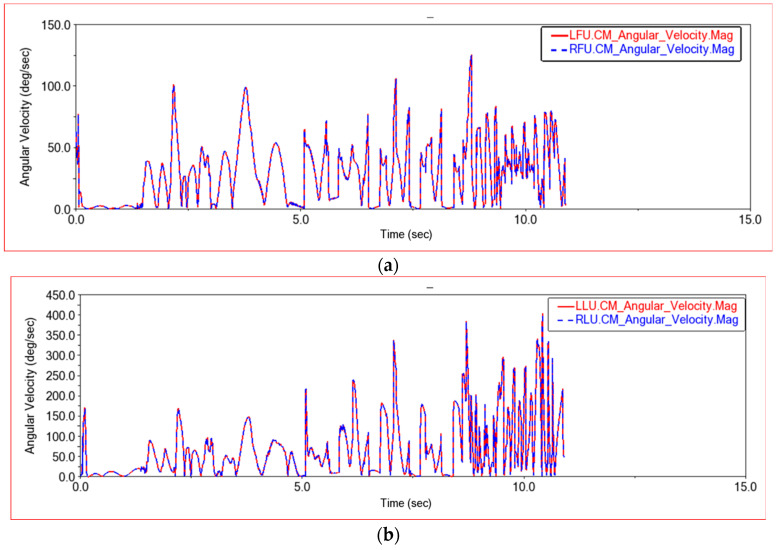
Angular speed comparison between front frame joint link and rear frame joint link. (**a**) Front frame joint link angular velocity change curve, LFU—Front frame upper left joint link, RFU—Front frame upper right joint link; (**b**) Rear frame joint link angular velocity change curve, LLU—Rear frame upper left joint link, RLU—Rear frame upper right joint link.

Figure 15 shows the angular velocity curve of the front frame joint link and the rear frame joint link in this experiment. On a flat road section, because the four wheels are driven at the same speed and move forward at a constant speed, the joint links of the front and rear frames remain relatively stationary. Therefore, on a flat road section, the angular velocity of the joint links is basically unchanged and remains at 0. When an obstacle is encountered, the body posture changes. In order to adapt to the terrain, the connecting rod rotates at an appropriate angle to change the body posture, and the angular velocity of the front and rear joint links changes. From Figure 15, it can be seen that the angular velocity curves of the joint links on the left and right sides of the front frame completely overlap, and the angular velocity curves of the joints on the left and right sides of the rear frame also completely overlap. It can keep synchronized changes and keep the body in a stable state. When encountering an obstacle, the angular velocity of the joint link of the rear frame is greater than the angular velocity of the joint link of the front frame. Because when encountering obstacles, the rear body will provide thrust for the front body, pushing the front body over the obstacle, the rear frame joint link will rotate more in the process of providing thrust to find the best thrust angle. Therefore, the angular velocity of the rear joint link is greater than that of the front joint link. From the above comparison chart, it can be seen that when the car climbs steps, the angular velocity changes more intensively. In order to adapt to the changes of each step of the steps and the bumps of the body when going up the steps, the joint link will rotate the angle and change the body posture, so the angular velocity changes frequently.

#### 4.2.3. Obstacle Crossing Experiment 2

**Figure 16:**
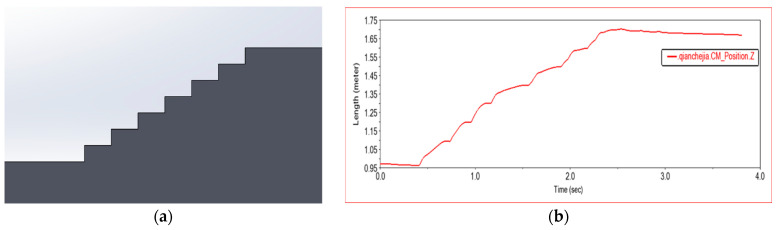

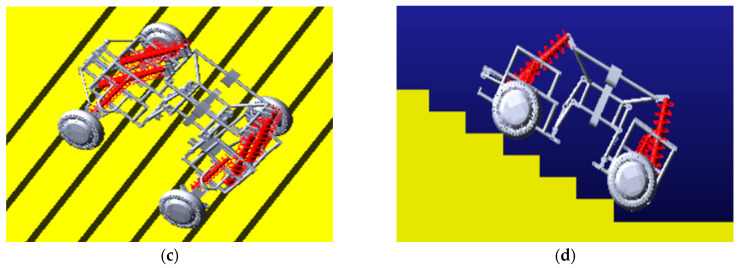
Experimental diagram in Experiment 2. (**a**) Experimental terrain, (**b**) Z-axis displacement of the center of mass of the front frame, (**c**,**d**) Simulation test process of car climbing stairs.

As shown in Figure 16, (a) is the three-dimensional terrain drawn by SolidWorks, which is composed of seven steps of 20 × 10 (cm), (b) is the change curve of the mass center of the front frame in the z-axis with time in the simulation experiment. It can be seen from the curve that the vehicle has undergone a total of seven ascents and then translated, which conforms to the characteristics of the terrain, and the curve changes smoothly. The vehicle moves smoothly when passing this step, which meets the movement requirements of climbing steps.

**Figure 17:**
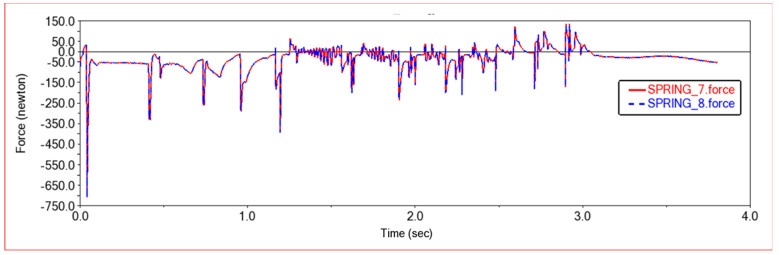
Force diagram of rear frame shock absorber in Experiment 2. SPRING-7-Rear frame left shock absorber; SPRING-8-Rear frame right shock absorber.

**Figure 18:**
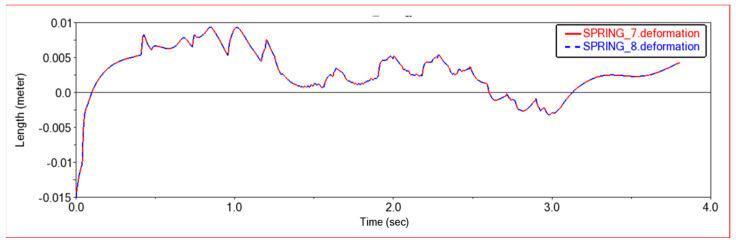
Deformation diagram of rear frame shock absorber in Experiment 2. SPRING-7-Rear frame left shock absorber; SPRING-8-Rear frame right shock absorber.

In Figure 17 and Figure 18, the force diagram and deformation displacement diagram of the left shock absorber of the rear frame and the right shock absorber of the rear frame in this experiment are shown. In terms of force and deformation, the left and right shock absorbers are exactly the same. This ensures the smooth movement of the body and the stability of the load bearing force. It can be seen from the force and deformation trends that the curve is mainly divided into three stages. The first stage is when the front wheels touch the steps and start to climb. At this time, the rear body must provide thrust to help the front wheels climb the steps, and the force of the two shock absorbers on the left and right of the rear frame increases. In the second stage, the rear wheels climb up the steps. At this time, the entire vehicle is on the steps, moving relatively smoothly, and the force of the rear frame shock absorber is relatively stable. The third stage is that the front wheels climb the steps and enter the plane motion stage. At this time, the front body will drag the rear body to climb the steps, and the force of the rear frame shock absorber will be reduced.

**Figure 19:**
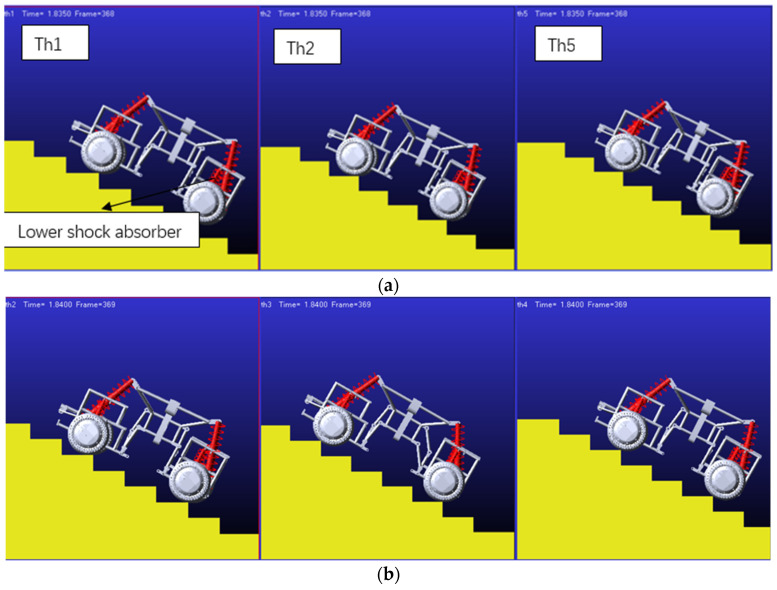
Vehicles with different stiffness shock absorbers climbing stairs. (**a**) Th1-Moderate stiffness, lower shock absorber, Th2-Soft stiffness, lower shock absorber, Th5-Stiffer stiffness, lower shock absorber; (**b**) Th2-Moderate stiffness, Upper shock absorber, Th3-Soft stiffness, Upper shock absorber, Th4-Stiffer stiffness, Upper shock absorber.

Shown in Figure 19 is the effect of climbing stairs when shock absorbers with different stiffness are used in robots; (a) is the effect diagram of climbing stairs when the stiffness of the lower four shock absorbers is adjusted under the condition that the upper four shock absorbers are kept at moderate stiffness. According to the simulation results, it can be obtained that the four lower shock absorbers play a supporting role for the robot, so the stiffness of these four shock absorbers needs to be larger. At the same time, when the shock absorber is too hard, the robot may jump when climbing stairs. Therefore, more research is needed to balance the robot’s load-bearing capacity and the stability when crossing obstacles. Furthermore, (b) is the effect diagram of climbing stairs when the stiffness of the lower shock absorber is moderate and the stiffness of the four upper shock absorbers is adjusted. According to the simulation results, when climbing stairs, the rear two upper shock absorbers will provide thrust. If the spring is too soft, in order to provide sufficient thrust, the rear link joint will increase the angle to adapt to terrain conditions and provide enough thrust to make it climb. Reasonable shock absorber configuration will improve the performance of the robot and the stability of obstacle crossing. Further research and optimization can be done.

**Figure 20:**
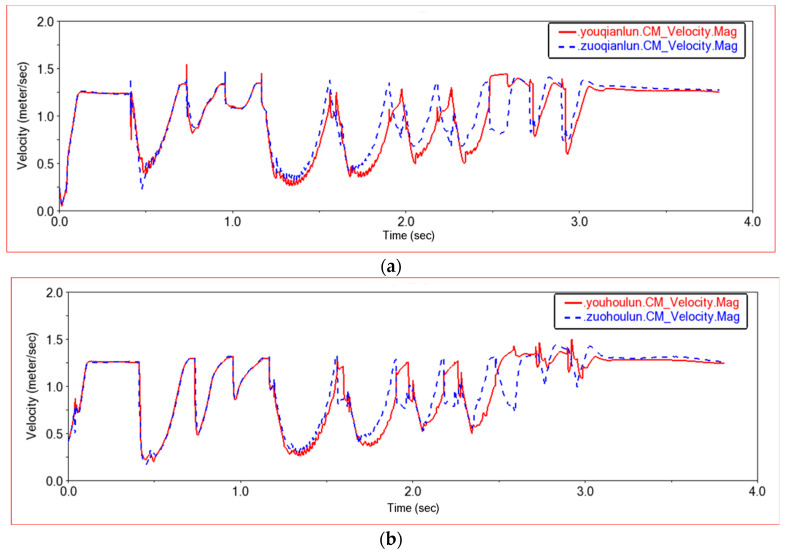
Wheel speed comparison curve in Experiment 2. (**a**) Front wheel speed curve, youqianlun.CM-Center of Mass of Right Front Wheel, zuoqianlun.CM-Center of Mass of Left Front Wheel; (**b**) Rear wheel speed curve, youhoulun.CM-Center of Mass of Right Rear Wheel, zuohoulun.CM-Center of Mass of Left Rear Wheel.

Shown in Figure 20 is a graph of the speed when four wheels pass through this step terrain. It can be seen from Figure 19 that the first three waveforms are completely overlapped between the left front wheel and the right front wheel, and the left rear wheel and the right rear wheel. At the beginning of the fourth wave, the left wheel and the right wheel have the same speed curve, but the movement of the right wheel lags behind the left wheel slightly. This is because the first three waveforms are the speed curves when only the front wheel climbs up the steps, and the fourth waveform is generated when the rear wheels climb up the steps. When the rear wheel climbs up the steps, the car will jump slightly. At this time, the front wheel is also on the step, and the right wheel slips, causing the right wheel to lag slightly behind the left wheel. Comparing the front wheel with the rear wheel longitudinally, it can be found that the speed curves of the front wheel and the rear wheel are almost the same, but in the first three waveforms, the speed of the rear wheel decreases more than that of the front wheel. This is because the first three waveforms are the speed curves when only the front wheels climb steps. When the front wheel encounters an obstacle, the speed of the front wheel decreases, and then the speed of the rear wheel also decreases. At the same time, when the rear wheel pushes the front wheel over the obstacle, the speed of the rear wheel must be further reduced. When the rear wheels also climb the steps, the entire car is on the steps, and the speed curves of the four wheels are basically the same. After the front wheel climbs the steps, the front wheel will drag the rear wheel to continue climbing the steps. When the rear wheel hits the step wall, the speed of the front wheel will decrease more than that of the rear wheel until the rear wheel also climbs the step; the speed of the four wheels will be the same again, and they will move in a plane.

**Figure 21:**
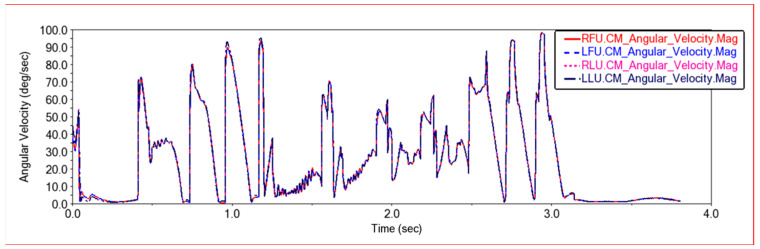
Angular speed comparison curve in Experiment 2. LFU—Front frame upper left joint link, RFU—Front frame upper right joint link, LLU—Rear frame upper left joint link, RLU—Rear frame upper right joint link.

Figure 21 shows the rotation angular velocity curve of the center of mass of the joint links on the front frame and the rear frame. It can be seen that the rotation angular velocity curves of the upper left joint link and the upper right joint link of the front frame, and the center of mass of the upper left joint link and the upper right joint link of the rear frame completely coincide. When climbing this step, the rotational angular velocity of the center of mass of the joint connecting rod is kept consistent, so that the car moves smoothly when climbing the step. It can be seen from the figure that in the process of climbing steps, when only the front wheel or the rear wheel is on the step, the rotational angular velocity of the center of mass of the joint link is relatively large. When the front and rear wheels are both on the steps, the car body structure is in a relatively stable state, and the rotational angular velocity of the joint link is small. However, because the car moves on the steps, there will be some slight jumps, so the frequency of angular velocity changes will increase.

#### 4.2.4. Obstacle Crossing Experiment 3

**Figure 22:**
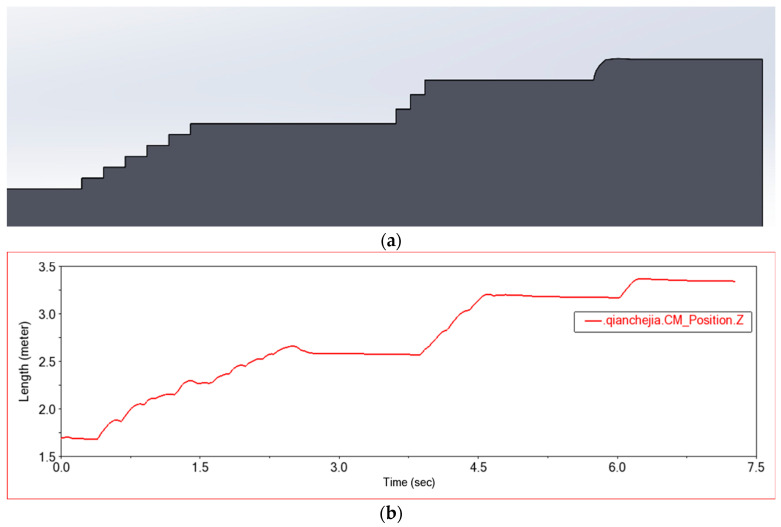

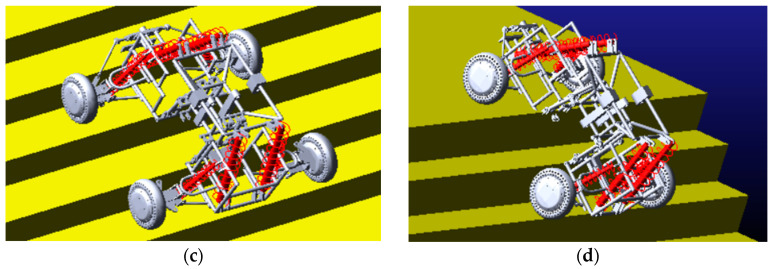
Experimental diagram in Experiment 3. (**a**) Experimental terrain, (**b**) Z-axis displacement of the center of mass of the front frame, (**c**,**d**) Simulation test process of car climbing stairs.

Shown in Figure 22a is a three-dimensional terrain created in SolidWorks, consisting of six steps of 30 × 15 (cm), three steps of 20 × 20 (cm), and one step of arc steps with a radius of 30 cm. Figure 22b shows the change curve of the front frame on the z-axis with time in this experiment. The curve is similar to the contour of the three-dimensional model, meets the design requirements, and the curve is smooth. When the car passes this obstacle, it runs smoothly, and the car has the off-road ability to break through high obstacles.

**Figure 23:**
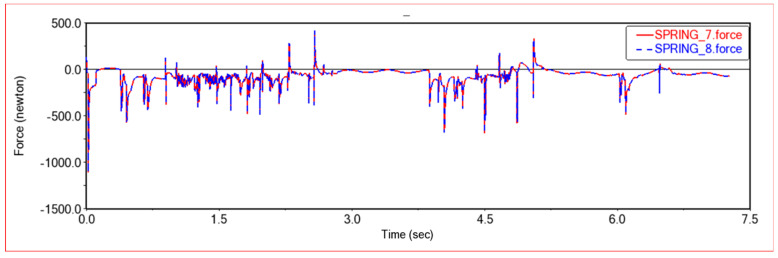
Force diagram of rear frame shock absorber. SPRING-7-Rear frame left shock absorber, SPRING-8-Rear frame right shock absorber.

**Figure 24:**
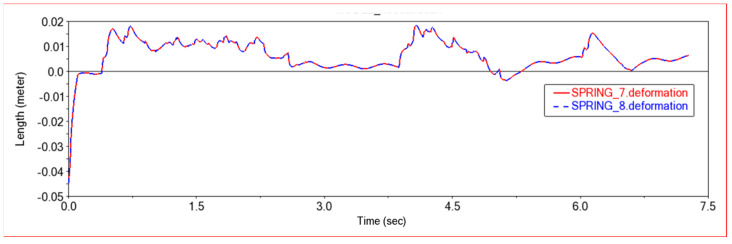
Deformation diagram of rear frame shock absorber. SPRING-7-Rear frame left shock absorber, SPRING-8-Rear frame right shock absorber.

Figure 23 and Figure 24 show the force curve and deformation curve of the two shock absorbers on the left and right of the rear frame in this experiment. It can be seen that the curves of the two shock absorbers are completely overlapped, indicating that the left and right shock absorbers can maintain synchronous force when climbing a high obstacle, so that the car body can climb over the obstacle smoothly. The first segment is the force curve when the car passes the sixth step of the 30 × 15 (cm) steps. When the front wheel climbs the first two steps, the vehicle body vibrates less, and when all four wheels are on the steps, the vehicle is more likely to vibrate when climbing the steps, and the shock absorber changes frequency faster. The middle section is the curve when the car passes the third-order 20 × 20 (cm) steps. Because the obstacles are higher, the thrust of the rear frame shock absorber is greater, and the frequency of change is also faster. The third segment is the force curve when the car passes through the arc step with a radius of 30 cm. When it touches an obstacle, the thrust increases, and then moves along the arc. The thrust decreases rapidly first and then decreases slowly, which conforms to the law of motion along the arc. Comparing the previous two experiments, it can be seen that when encountering a high obstacle, the shock absorber of the rear frame will deform and provide greater thrust to make the car climb up the obstacle. When all four wheels are on the steps, as the height of the steps increases, the thrust of the shock absorber of the rear frame will increase, and when the steps are increased, jumping is more likely to occur in the process of climbing the steps.

**Figure 25:**
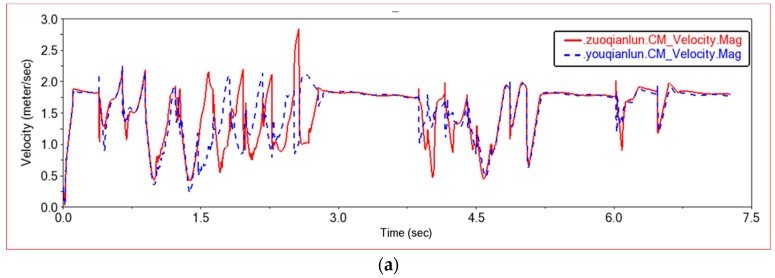

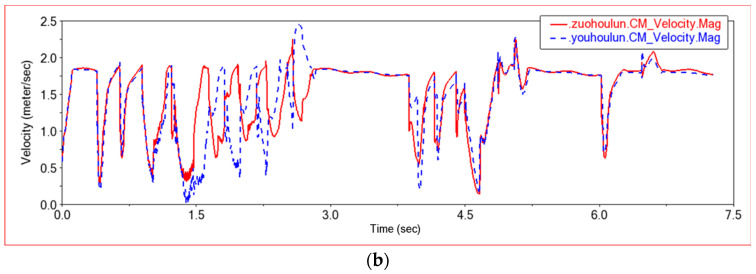
Wheel speed comparison curve in Experiment 3. (**a**) Front wheel speed curve, youqianlun.CM-Center of Mass of Right Front Wheel, zuoqianlun.CM-Center of Mass of Left Front Wheel; (**b**) Rear wheel speed curve, youhoulun.CM-Center of Mass of Right Rear Wheel, zuohoulun.CMCenter of Mass of Left Rear Wheel.

Figure 25 shows the speed curve of the front wheel and the rear wheel in this experiment. The speed curves of the four wheels are similar, but when encountering obstacles, the speed of the rear wheels decreases more than that of the front wheels. Because the rear wheels need to provide thrust when encountering steps, the front wheels are pushed to climb the steps, and the speed of the rear wheels decreases to provide greater thrust. When the entire body climbs up the high steps, the climbing process will be more difficult. The body will tilt to change the climbing posture. The left wheel first climbs the steps and then the right wheel until the rear wheels have also climbed the steps. When the front wheel finishes climbing the steps, the rear body will be dragged to climb the steps, and the speed drop of the rear wheels will decrease. When the rear wheels leave the steps, the body will jump because of inertia. By comparing 15 cm steps and 20 cm steps, it can be seen that the speed will decrease when climbing 20 cm steps. Comparing the low obstacle experiment, the medium obstacle experiment and the high obstacle experiment, the car needs more driving force when passing high obstacles. The speed curve is smoother when the obstacle is low, and the speed curve is sharper when the obstacle is high. When passing high obstacles, the speed change range increases. When climbing a low step, the left and right wheels are synchronized. When climbing a high step, the body posture will change, so that the left wheel will go up a step first, and the right wheel will go up again, moving upward step by step.

**Figure 26:**
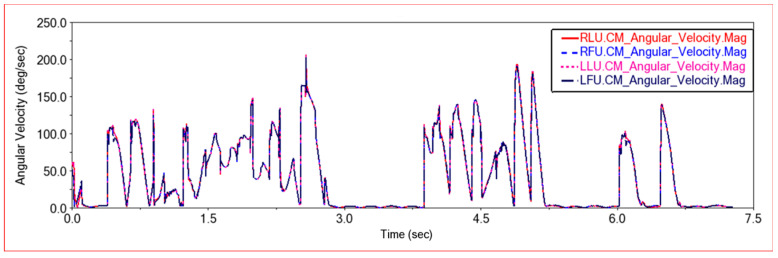
Angular speed comparison curve in Experiment 3. LFU-Front frame upper left joint link, RFU-Front frame upper right joint link, LLU-Rear frame upper left joint link, RLU-Rear frame upper right joint link.

Shown in Figure 26 is the rotation angular velocity curve of the center of mass of the joint connecting rods on the front frame and the rear frame in this experiment. The rotation angular velocity of the center of mass of the left and right joint links of the front frame and the left and right joint links of the rear frame completely coincide. It can be seen that the posture of the middle body can be kept relatively stable when passing through high steps. By comparing with the previous two experiments, it can be seen that when the vehicle is climbing steps, the trend of the rotation angular velocity of the center of mass of the joint link changes. When only the front wheel is on the step, the rotational angular velocity increases. When both the front and rear wheels are on the step, the rotational angular velocity decreases and remains relatively stable. When the front wheel leaves the step and only the rear wheel is on the step, the rotational angular velocity again increases. After the rear wheel leaves the step, the rotational angular velocity drops to zero. As the height of the steps increases, the rotational angular velocity of the center of mass of the joint connecting rod also increases, and the amplitude of change increases. When the entire vehicle is located on the steps, the climbing method of the high steps has changed, and more deformation is required to adapt to the environment of the steps. The car still needs a large link rotational angular velocity on the steps, so the rotational angular velocity of the center of mass of the joint link decreases more on the low steps than on the high steps.

Through simulation and analysis, compared with similar robot structures proposed in previous scientific literature, the following characteristics and performance improvements can be seen. (i) Compared with the eight-wheeled robot proposed in the literature [13], which uses the suspension system to realize the deformation of the body, this paper adopts the four-wheeled robot to realize the deformation of the body, and the structure design is simpler. (ii) Compared with the automatic stair climbing robot proposed in document [14], this robot can cope with more complex obstacle environments, and has strong carrying capacity, which can complete more tasks. (iii) Compared with the literature [16], this paper adopts a common independent suspension system, which has a simple structure and low cost. With the designed link-spring joint system, the robot can better adapt to the terrain.

## 5. Ansys Analysis

In the above experiments, the Adams software is used to simulate the vehicle bearing performance and obstacle-crossing performance, which meet the design requirements. Through the analysis of the vehicle structure, it can be known that the weakest part of the vehicle should be the connection between the wheels and the upper and lower suspensions when carrying the load. This is the transition link between the vehicle and the wheels, which is the most concentrated force of the vehicle. The finite element analysis of the structure is carried out using ANSYS software to obtain the stress and strain here, which can more scientifically evaluate the bearing capacity of the vehicle.

According to the design requirements, the vehicle needs to be able to withstand a mass of 200 kg, so the vehicle can withstand a pressure of 2000 N. The finite element analysis of the connection between the wheel and the suspension is carried out. Through the simulation experiment with the above Adams software, the stress of each moving pair of the wheel connection can be obtained as the boundary condition of the finite element simulation when the vehicle carries 2000 N, as shown in Table 2 and Table 3.

### 5.1. Finite Element Analysis of Front Wheel Connector

**Figure 27:**
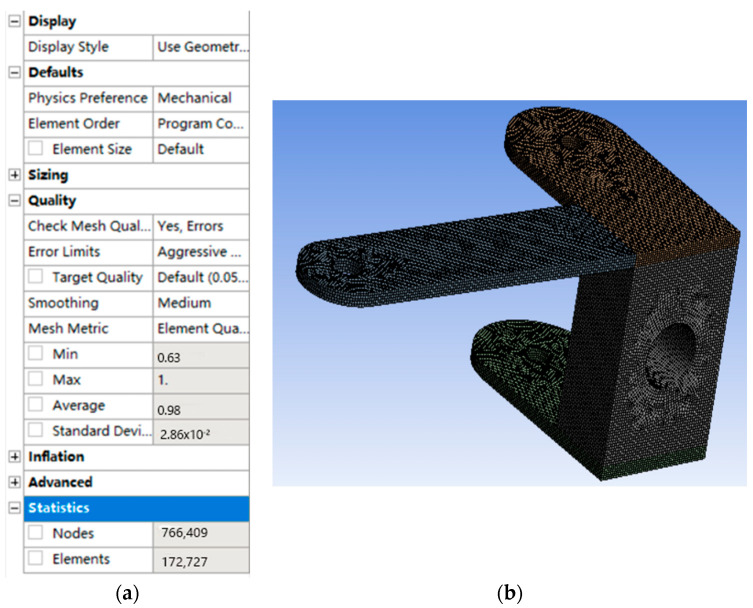
Mesh division and mesh quality of front wheel connector. (**a**) Element quality of the mesh, (**b**) Effect picture of meshing.

In this paper, the finite element analysis of the front and rear wheel connector is carried out by WorkBench2021 R2 to verify the rationality of the design. Firstly, the finite element analysis of the front wheel connector is carried out, and the three-dimensional model of the front wheel connector drawn in SolidWorks is imported into the geometric structure of static structure. The imported three-dimensional model is segmented in the geometric structure to facilitate meshing. The multi-region method is used for meshing, and the final mesh effect is shown in Figure 27. The mesh quality is 0.98084, which meets the mesh requirements for finite element analysis.

**Figure 28:**
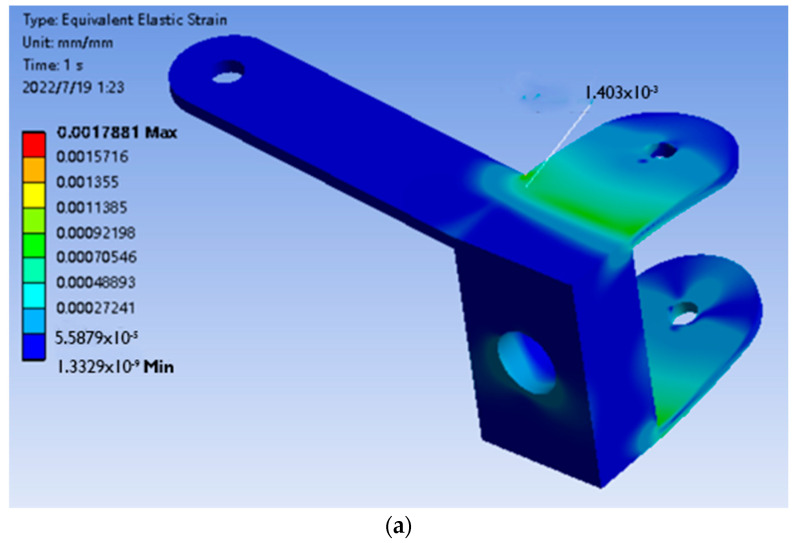

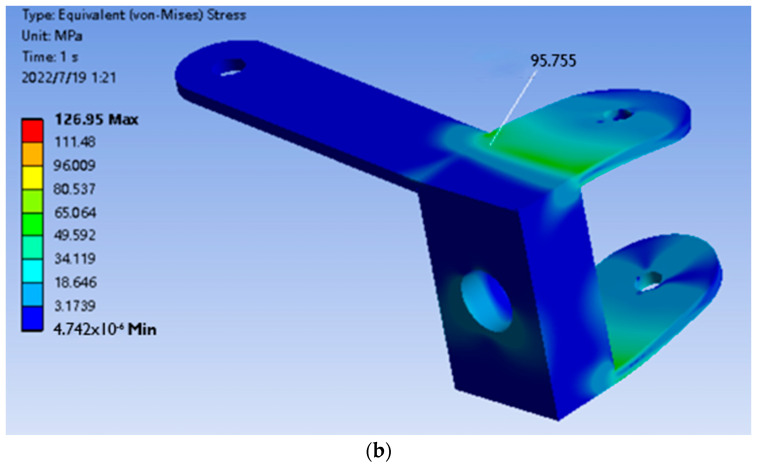
Finite element analysis result graph of front wheel connector. (**a**) Equivalent strain result graph; (**b**) Equivalent stress result graph.

After the mesh is divided, the force conditions of the front wheel connectors are applied to the corresponding positions according to the table for solution. The stress and strain of the front wheel connector under load are obtained, as shown in Figure 28:

It can be seen from the above figure that the maximum area of stress and strain occurs on the plane in contact with the horns, and the maximum appears at the right corner on the back. The maximum strain is 1.4 × 10^−3^ mm, the maximum stress is 95.755 MPa.

According to material mechanics, the formula [43] for the safety factor of the workpiece is

(10) $$n=\frac{\sigma_{s}}{\sigma_{\max}}$$

In the formula: $\sigma_{s}$ is the material yield limit, $\sigma_{\max}$ is the maximum equivalent stress. When n > 1, it indicates that the strength meets the requirements, otherwise the structure will be destroyed due to insufficient strength. According to the literature, the safety factor of automobile parts is generally 1.5 [44]. According to the literature, the tensile yield strength of aluminum alloy is 280 MPa. The safety factor of the front wheel connection can be obtained as $n=\frac{280}{95.755}=2.92$ > 1.5, which meets the strength requirements.

### 5.2. Finite Element Analysis of Rear Wheel Connector

**Figure 29:**
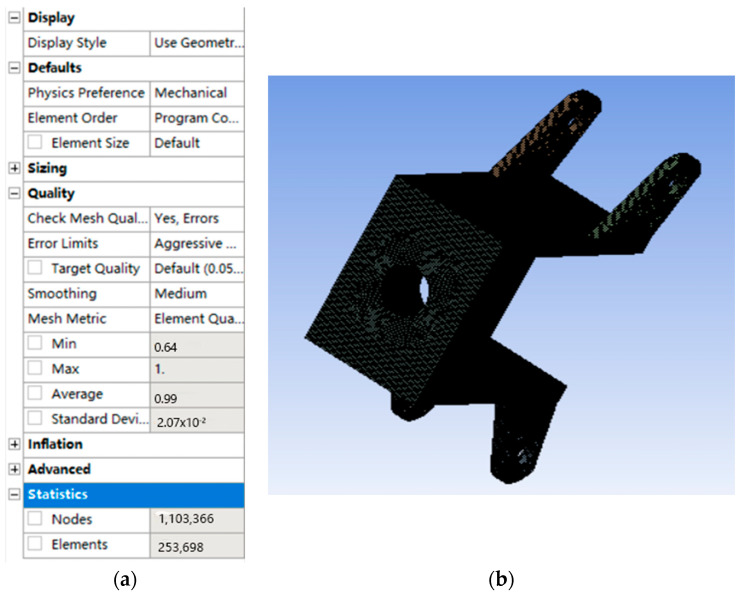
Mesh division and mesh quality of rear wheel connector. (**a**) Element quality of the mesh, (**b**) Effect picture of meshing.

The rear wheel connectors are meshed in the same way as the front wheel connectors. The mesh effect obtained is shown in Figure 29, and the mesh quality is 0.99251, which meets the mesh requirements for finite element analysis.

**Figure 30:**
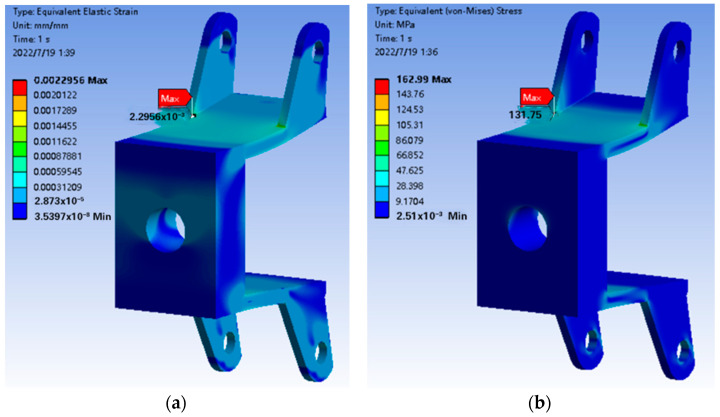
Finite element analysis result graph of rear wheel connector. (**a**) Equivalent strain result graph, (**b**) Equivalent stress result graph.

After the mesh is divided, the load in Table 2 is applied to the corresponding position of the rear wheel connector to solve it. The strain and stress of the rear wheel connection under the load condition are obtained, as Figure 30 shows:

From the stress and strain map above, it can be seen that the stress and strain are mainly concentrated on the upper beam. The maximum value is at the junction of the upper beam and the left ear, the maximum value of strain is $2.2956\times 10^{-3}$ mm, the maximum stress is 131.75 MPa. According to the formula, the safety factor of the rear wheel connector can be obtained as $n=\frac{280}{131.75}=2.125$ > 1.5, which meets the strength requirements.

## 6. Real Vehicle Test and Analysis

**Figure 31:**
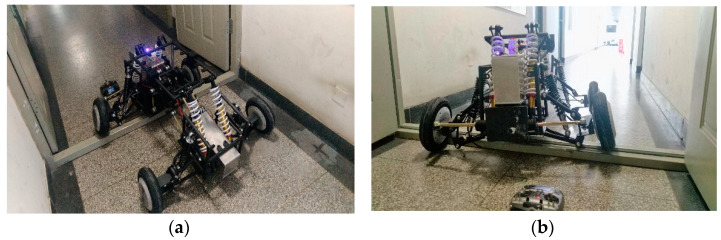
Vehicle crossing beam test. (**a**) Top view of vehicle crossing beam (**b**) Front view of vehicle crossing beam.

**Figure 32:**
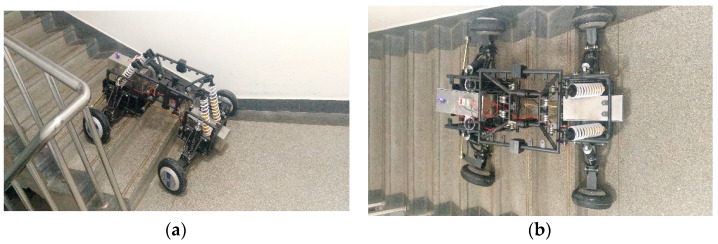
Vehicle climbing stairs experiment. (**a**) Side view of vehicle climbing stairs (**b**) Top view of vehicle climbing stairs.

The designed car body structure is processed in the factory and the assembly of the whole vehicle is completed in the laboratory. The control system is built with STM32, the radio link AT10 remote controller is used, and the R9DS receiver is used to receive the SBUS signal of the remote controller, so as to realize the purpose of using the remote controller to control the robot movement. Under limited experimental conditions, the experiment of the robot crossing the ground beam obstacles and climbing stairs was carried out. The experimental results are shown in Figure 31 and Figure 32.

Through experimental tests, the robot can pass through the ground beam obstacles and complete the task of climbing stairs. The experimental results show that the mechanical structure design is reasonable and consistent with the previous simulation results. Meanwhile, during the experiment, the friction between the wheel and the ground is also the key for the robot to climb the stairs smoothly. When the friction between the wheel and the ground is small, the robot will slip when climbing the stairs, which is consistent with the Adams simulation that when the robot passes through higher steps, it is necessary to set a larger friction coefficient. It can be seen from the experimental results that when climbing stairs, it is necessary to select rough tread and increase the friction between the tire and the ground, which can have a better effect.

## 7. Conclusions

In this paper, an all-terrain adaptive five DOF robot was designed, which adopts modular design ideas and combines them with linkage joints. Then, the mathematical model was established and the forward and inverse kinematics were analyzed. Furthermore, Adams software was used to carry out load-bearing experiment and obstacle-crossing experiment analysis. Through the analysis of the curve generated by the simulation experiment, the results show that the structure design can carry 2000 N. When facing continuous obstacles, it can pass smoothly, and it can also complete traversal when encountering high obstacles with good motion stability. Through theoretical mechanics analysis, it can be concluded that the wheel connector is a key part of the car. Ansys software was used to perform finite element analysis on the wheel connector to verify that it meets the strength design requirements. It can be seen that this design is feasible. Through real vehicle experiments, the obstacle surmounting performance of the robot is tested, and the conditions for the robot to climb stairs are obtained. This aim of this article was to design a wheeled cross-country robot; a block structure design is proposed, which uses the joint system composed of connecting rods to establish connections, so that the robot has higher flexibility. Using this idea, the legged robot can also be improved by further improving its ability to overcome obstacles.

## Figures and Tables

**Table 1:** D-H parameters table.

$i_{-1-i}$	$\theta_{i}\;({}^{\circ})$	$d_{i}\;(\mathrm{mm})$	$a_{i}\;(\mathrm{mm})$	$\alpha_{i}\;({}^{\circ})$
r–0	$\theta_{0}$	0	0	0
0–1	$\theta_{1}$	0	$a_{1}$	0
1–2	$\theta_{2}$	0	$a_{2}$	0
2–3	$\theta_{3}$	0	$a_{3}$	0

**Table 2:** Force on moving pair of front wheel connector (N).

Kinematic Pair	$F_{x}$	$F_{y}$	$F_{z}$	Resultant Force
Upper rotating pair	0	185	−500	533.128
Middle rotating pair	−500	0	0	500
Lower rotational pair	0	−150	550	570.088
Upper plane pair	600	0	0	600
Lower plane pair	−600	0	0	600

**Table 3:** Force on moving pair of rear wheel connector (N).

Kinematic Pair	$F_{x}$	$F_{y}$	$F_{z}$	Resultant Force
Left upper rotational pair	0	325	−20	325.615
Right upper rotation pair	0	325	−20	325.615
Middle rotating pair	600	0	0	600
Left lower rotational pair	0	−325	−275	425.735
Right lower rotational pair	0	−325	−275	425.735
